# A Review on Genetics of Sleep Disorders

**Published:** 2012

**Authors:** Reza Bidaki, Mina Zarei, Ali Khorram Toosi, Mitra Hakim Shooshtari

**Affiliations:** 1 Department of psychiatry Rafsanjan University of medical sciences, Rafsanjan, Iran.; 2 Tehran University of Medical Science. Tehran, Iran; 3 Assistant professor of Child and adolescence psychiatry. Department of psychiatry, Tehran University of medical sciencs. Tehran, Iran.

**Keywords:** Gene, Genetic study, Sleep disorders

## Abstract

One-third of population deal with sleep disorders which might be due to social, economic or medical problems. Studies on twins have indicated the role of genetic factors in these disorders. Monozygotic twins have a very similar hypnogram. A higher prevalence of some sleep disorders is reported in relatives of the patients with these disorders. Genes also affect sleep disorders as well as some other disorders at the same time. Sleep disorders can also influence the level of the personal and social functioning. Recent genetic advances have clarified the role of different genes in sleep disorders. The purpose of this article is to present a brief review about the role of genetic factors in some of the sleep disorders.

## Introduction

One-third of population deal with sleep disorders which might be due to social, economic or medical problems (1). Genetic predisposing factors are among the ones which necessitates further research to elucidate their role in sleep disorders. Most of the current progress in the study of sleep genetics comes from animal models (dogs, mice, and drosophila) (2). Sleep has been observed in all vertebrates studied and in several invertebrates, notably the fruit fly Drosophila melanogaster. In all species, a substantial portion of life is spent in this behavioral state and disturbed sleep or lack of sleep has immediate negative impacts on performance and health (3). The heritability of sleep patterns has been shown in studies of monozygotic twins, and sleep electroencephalogram patterns offer a unique genetic fingerprint which may assist in the identification of genes involved in the regulation of sleep (4, 5). Recent genetic advances have clarified the role of Hypocretin/ Orexin System in sleep disorders (6). Although it is agreed upon that sleep fulfills a fundamental biological need, the function of sleep remains an enigma (3).

Genetic factors have also effects on normal sleep. Monozygotic twins have a very similar hypnogram. Specially their sleep cycle periods, rapid eye movement (REM) manifestations, and sleep latency times are very similar to each other (7).

Most sleep disorders result from complex interactions between genes and the environment (8). Recent progress in molecular genetics and the development of detailed human genome map have already led to the identification of genetic factors in several complex disorders (2). Disorders such as enuresis, restless leg syndrome (RLS), sleep apnea syndrome, parasomnias, periodic limb movement syndrome (PLMS) are among sleep disorders which are associated with genetic factors (6, 9). At one extreme are the disorders with simple Mendelian patterns of inheritance such as familial advanced sleep phase syndrome, and at the other extreme are diseases such as insomnia, which can be associated with a multitude of medical and psychiatric conditions (10).

This brief review will present the role of genetic factors in some of the sleep disorders.

## Materials and Methods

Articles published in the past ten years were searched on PubMed (MEDLINE) using the keywords "sleep disorders" and "genetics". Abstracts were then analyzed and a brief review was prepared about the role of genetics in sleep disorders such as circadian rhythm disorder, fatal familial insomnia (FFI), familial advanced sleep phase syndrome (FASPS), narcolepsy, insomnia, obstructive sleep apnea/hypopnea syndrome (OSAHS), restless leg syndrome, depression, sleep related epilepsy and headache, sleep walking, nocturnal enuresis, idiopathic hypersomnia, caffeine-related sleep disorders, Klein-Levin syndrome (KLS), and hypoventilation/ hypoxia.

## Results

Based on the results of analyzing the articles which were found, only few sleep disorders have an established genetic basis including four rare diseases that may result from a single gene mutation: fatal familial insomnia, familial advanced sleep-phase syndrome, chronic primary insomnia, and narcolepsy with cataplexy. However, most sleep disorders are complex in terms of their genetic susceptibility together with the variable expressivity of the phenotype even within a same family (5).

In this article we briefly will review some of these sleep disorders. 

*Insomnia*

Insomnia is defined as a complaint of difficulty in initiating sleep, difficulty in maintaining sleep, waking up too early, or sleep that is chronically non-restorative or poor in quality (9).

Insomnia is a serious health problem that affects millions of people. Population-based surveys have estimated the prevalence of insomnia to be about 10% to 50% in general population (11).

Approximately 35% of people with insomnia have a positive family history, with the mother being the most commonly affected family member. Still, because so many factors are involved in insomnia, a genetic component is difficult to define (12).

*Insomnia due to mental disorder*

Insomnia due to a mental disorder is the most common diagnosis among individuals presenting to a sleep center for evaluation and treatment of chronic insomnia (13).

*Major depressive disorder*

Insomnia associated with major depressive disorder includes normal sleep onset but repeated awakenings during the second half of the night and premature morning awakening (14).

It seems that association between sleep and depression has a genetic source. The results of the study conducted by Gregory and colleges which evaluated the association between sleep problems and depression symptoms in twins aged 8 and 10 years revealed that there is an association of 30% in 8-year-old and of 11% in 10-year-old children between sleep disorders and depression (15).

*Psychophysiologic insomnia*

Psychophysiologic insomnia is also referred to as learned or conditioned insomnia. A patient with psychophysiologic insomnia is often over-focused on the problem of sleep and experiences increased arousal at bedtime when preparing to sleep (16, 17).

*Adjustment insomnia*

Often referred to as acute insomnia, adjustment insomnia has one-year prevalence among adults of 15 to 20%. It is more common in women than men, and in older adults rather than younger adults and children (17). The essential feature to a diagnosis of adjustment insomnia is the presence of an identifiable stressor. It is typically of short duration (days to weeks), lasts no more than three months, and is expected to resolve with either adaptation to or resolution of the stressor. Should the patient’s symptoms persist more than 3 months, an alternate diagnosis of one of the more chronic insomnias should be considered.

*Hypersomnia*

*Idiopathic Hypersomnia*

It is diagnosed when no other cause can be found for excessive somnolence occurring for at least 1 month (14). It is considered either monosymptomatic, manifested only by excessive daytime sleepiness and not by abnormal awakening, or polysymptomatic, characterized by excessive daytime sleepiness, nocturnal sleep of abnormally long duration, and signs of “sleep drunkenness” on awakening (18). It is defined by severe and continuous drowsiness and an autosomal dominant pattern is proposed for it (19).

*Narcolepsy*

Narcolepsy is characterized by two major symptoms, excessive daytime sleepiness (EDS) and cataplexy and other manifestations of abnormal REM sleep (20).

Narcolepsy is a disabling sleep condition and research has revealed the complexity of underlying genetic and environmental influences in the development of this disorder (4).

Close relatives have about 1-2% probability of narcolepsy, a rate of 10-40 folds higher than normal population (21). 

The disorder has the highest association with Human Leukocyte Antigen (HLA) DR2/DQW1 (22). For the first time in 1983 in Japan, narcolepsy was associated with HLA-DR2 and after that, association with DR2, DR5 and then DRB1*1501/DRB1*1503 were also determined (23). Other studies indicated that DQB1*0602 is a better marker for narcolepsy, especially in African Americans (24).

Sixty-eight percent of narcoleptic pro-bands have HLA-DRB1*15 and HLA-DQB1*602. In some families, in monozygotic twins with cataplexy, HLA-DR14 (DW) DQB1*0503 and DR4 (DW4)/DQB1*0302 have been documented (22).

Narcolepsy might exist in absence of HLA-DRB1*15/DQB1*0602. It is estimated that 88 to 98% of patients who suffer from cataplexy are HLA-DQB1*0602 positive. DRB1 and DQB1 genes have also been implicated in narcolepsy but no mutations have been found in them (22).

In one study, binding to locus in 5mb region of chromosome 21q is demonstrated (4, 20).

Genetic studies in an autosomal recessive canine model of narcolepsy and in gene-targeted mice have identified the hypothalamic hypocretin (orexin) neuropeptide system as a key target for human narcolepsy (8, 21).

Hypocretin level in the cerebrospinal fluid (CSF) of these patients is low (25).

Narcolepsy is shown in animals such as Deberman/Labrador dogs in which mutation in hypocretin receptor 2 has been determined (26).

*Klein-Levin Syndrome (KLS)*

KLS are characterized by recurrent hypersomnia and abnormal feeding behaviors (27, 28). This disorder is more likely to happen in the people with HLA-DQ-B1 and DQ-D2 (29, 30).

*Circadian Rhythm disorder*

The term "circadian" refers to a time frame of "about 1 day" and captures an interesting feature of the circadian clock, namely, that it runs slightly longer or shorter than 24 hour (31).

Circadian rhythm disorder includes a wide range of conditions involving a misalignment between actual and desired sleep periods (14).

The clock gene was identified in 1990. This gene's mutation in homozygote mice caused an extension of night and day course to 28 hours for them (24). Day-night circadian rhythm is regulated by cells which are dependent on cryptochrome (CRY) and period (PER) genes activity. These genes regulate CRY and PER protein levels (32). The genetic basis of circadian rhythms has been explored using Drosophila and rodent models (8).

Circadian rhythm disorder follows an autosomal dominant pattern with a polymorphism in hPER2 alkylamine, ran-acetyltransferase and HLA genes. The first gene three alleles of which recognized in circadian rhythm named PER was found in 1971 in Drosophila (33).

CRY and PER protein levels inhibit their genes. This inhibition increases transcription of genes that bind clock and BMAL1 proteins to E-box individuals. This stimulates some areas on CRY and PER genes. Cross reaction between circadian rhythm proteins might decrease the feedback course (34).

PER phosphorylation by creatine kinase inhibitor (CKI) might cause its dispersion (35). It seems that BMAL1 transcription is via REU-ERBα which occurs through clock-BMAL1 binding to E-box components. It is believed that histone acetylation plays a role in circadian rhythm formation (24, 35).

*Fatal Familial Insomnia (FFI)*

In 1986, FFI in which gene mutation plays a role was introduced by Gomarcia. Progressive decrease in sleep period, lack of sleep with slow waves and non-unity of periodic sleep are the characteristics of this disorder (36).

This neuro-genetic disorder is due to mutation in codon D178N of prion protein (PrP) gene which is the cause of special thalamic nuclei degeneration and has been the predominant mutation found in nearly all families with FFI (37).

The inheritance pattern of this disorder is autosomal dominant, with same sexual relation and high penetration. The mutation is the result of change in aspartate location (38).

Patients who have homozygote methionine in codon 129 show a shorter period of disease compared with those who have heterozygote valine-methionine in codon 129 (38).

*Familial Advanced Sleep Phase Syndrome (FASPS)*

FASPS is an inherited abnormal sleep pattern in which the individual is a "morning lark" and consistently goes to sleep very early and wakes up very early as well. The individual's blood melatonin level and the body core temperature rhythm which are preordained by human daily biologic (circadian) clock are phase-advanced by 3 to 4 hours (39).

Recent studies have shown that mutations in human period 2 gene (hPER2) are associated with autosomal dominant FASPS (8, 21). Mutation in location 2106 (A-G) of epsilon casein kinase 1 (CK1ε)-binding herp2 gene on chromosome 2 results in translocation of serine in amino-acid 662 with a glycine (56624) (40).

*Obstructive Sleep Apnea/Hypopnea Syndrome* *(OSAHS)*

It is characterized by periods of functional obstruction of the upper airway during sleep, resulting in decreases in arterial oxygen saturation (14). OSAHS is a polygenetic disorder with a complex phenotype (9, 41). The contribution of genetic factors to obstructive sleep apnea syndrome (OSAS) has led to a better understanding of this complex disorder that may be part of a larger syndrome associated with respiratory, cardiovascular, and metabolic dysfunction (8, 21).

One study in Japan illustrated the association between HLA-DR2 and OSAHS (42). Also, in an American study on narcoleptic population with OSAHS, the frequency of HLA-DR2 sequence in such patients was higher than normal subjects (43).

*Restless Leg Syndrome*

It is an uncomfortable, subjective feeling of the limbs that also known as Ekbom syndrome (14). More than half of the cases have a familiar pattern. Its risk is 3-6-fold more in close relatives. An autosomal dominant pattern has been suggested for the disorder (7, 9).

*Sleep Related Epilepsy*

Sleep disorders can exacerbate seizure (44). It can be genetically inherited (45, 46). There is no Mendelian transfer model in these cases (9, 17).

*Sleep Related Headache*

It often appears during sleep and is marked by an on-off pattern of attacks (14).

Around 80% of these patients have family histories of migraine. Familial hemiplegic migraine is inherited via autosomal dominant pattern and several mutations occur on chromosomes 19 and 1 (45). Although there is not a strong familial pattern in cluster headache, their close relatives suffer the headache 7-fold more than normal population (9, 19).

*Sleep Walking (Somnambulism)*

This disorder consists of a sequence of complex behaviors that are initiated in the first third of the night during deep Non-REM sleep (45). A strong genetic pattern is suggested for this dysfunction (46). The rate of sleep walking in a child whose none of the parents are involved is 22% and if one or both of the parents are involved, this rate will increase to 45% (47). Sleep walking is associated with HLA O501 and DQ-B1 (45, 46).

*Nocturnal Enuresis*

It is involuntary urination during sleep after a certain age when there should be control over bladder (48). Four gene locations including 8q, 12q, 12qh, and 22qu are found for this problem (49).

*Substance- induced sleep disorder*

Any sleep disturbance can be caused by a substance (14).

*Caffeine-Related Sleep Disorders*

A prominent disturbance in sleep that is sufficiently severe to warrant independent clinical attention (14). High caffeine intake and intoxication, and also its withdrawal are related to a group of genetic factors (50). A probable effect of two genes located on chromosome 17q is proposed (51). Results of linkage studies have also demonstrated the relation between 2q chromosome regions in sleep disorders associated to caffeine (50, 51).

*Sleep-related Hypoventilation/Hypoxia*

It refers to several conditions marked by impaired ventilation in which the respiratory abnormality appears only during sleep (14). Sleep disorders because of hypoventilation/ hypoxia due to inferior airways obstruction might be caused by α1-antithripsin enzyme deficiency which is a genetic disorder with about one in 3,000 occurrence (51, 52). Cilia motivation dysfunction syndrome is also a genetic disorder which might cause bronchiectasis (9). Musculoskeletal disorders might also be transferred genetically and cause sleep disorders due to hypoventilation (7).

## Conclusion

Although most of the sleep disorders do not have by now an identified molecular basis, both animal models and modern techniques are being increasingly applied to determine the contribution of genes to sleep and its associated disorders (4, 53, 54). These discoveries have very clinical importance because they will provide clues to understand pathogenesis of sleep disorders, to assess the risk for diseases before symptom onset and also to find new drug targets to treat and to prevent the underlying conditions.

## Authors’ contributions

RB conceived and designed the evaluation and revised the manuscript. MZ searched and collected the data, interpreted them and drafted the manuscript. AKT and MHS conceived and designed the evaluation. All authors participated in data analysis, read and approved the final manuscript. 

## Notes

**Declaration of interest:** None.

## References

[1] Risch N, Merikangas K (1996). The future of genetic studies of complex human diseases. Science.

[2] Dauvilliers Y, Maret S, Tafti M (2005). Genetics of normal and pathological sleep in humans. Sleep Med Rev.

[3] Franken P, Tafti M (2003). Genetics of sleep and sleep disorders. Front Biosci.

[4] Tafti M (2009). Genetic aspects of normal and disturbed sleep. Sleep Med.

[5] Dauvilliers Y, Tafti M (2008). The genetic basis of sleep disorders. Curr Pharm Des.

[6] van Loo KMJ, Martens GJM (2007). Genetic and environmental factors in complex neuro-developmental disorders. Curr Genomics.

[7] Kimura M, Winkelmann J (2007). Genetics of sleep and sleep disorders. In: Cellular and Molecular Life Sciences. Cell Mol Life Sci.

[8] Taheri S (2004). The genetics of sleep disorders. Minerva Med.

[9] American Academy of Sleep Medicine (2005). International classification of sleep disorders: diagnostic and coding manual.

[10] Raizen DM, Mason TBA, Pack AI (2006). Genetic basis for sleep regulation and sleep disorders. Semin Neurol.

[11] Carson S, Thakurta S, Yen P (2008). Drug class review: Insomnia.

[12] Bastien CH, Morin CM (2000). Familial incidence of insomnia. J Sleep Res.

[13] Buysse DJ, Reynolds CF 3rd, Kupfer DJ, Thorpy MJ, Bixler E, Manfredi R (1994). Clinical diagnoses in 216 insomnia patients using the international classification of sleep disorders (ICSD), DSM-IV and ICD-10 categories: a report from the APA/NIMH DSM-IV Field Trial. Sleep.

[14] Benjamin JS, Virginia AS (2007). Synopsis of psychiatry.

[15] Gregory AM, Früuhling V, Rijsdijk, Jennifer Y, Lau F, Ronald E, Dahl, Thalia C, Eley (2009). The direction of longitudinal associations between sleep problems and depression symptoms: A study of twins aged 8 and 10 years. Sleep.

[16] Tsuno N, Besset A, Ritchi K (2005). Sleep and depression. J clin psychiatry.

[17] Summers MO, Crisostomo MI, Stepanski EJ (2006). Recent developments in the classification, evaluation, and treatment of insomnia. Chest.

[18] Michel Billiard (1996). IDIOPATHIC HYPERSOMNIA. Neurologic Clinics.

[19] Breslau N, Roth T, Rosenthal L, Andreski P (1996). Sleep Disturbance and Psychiatric Disorder: A longitudinal epidemiological study of young adults. Bio Psychiatry.

[20] Aldrich MS (1999). Narcolepsy. Neurology.

[21] Billiard M, Pasquié-Magnetto V, Heckman M, Carlander B, Besset A, Zachariev Z (1994). Family studies in narcolepsy. Sleep.

[22] Hayduk R, Flodman P, Spence MA, Erman MK, Mitler MM (1997). HCA hHaplotypes, polysomnography, and pedigrees in a case series of patients with narcolepsy. American Sleep Disorders Association and Sleep Research Society.

[23] Mignot E (1998). Genetic and familial aspects of narcolepsy. Neurology.

[24] Mignot E, Kryger MH, Roth T, Dement WC (2000). Pathophysiology of narcolepsy. Principles and Practice of Sleep Medicine.

[25] Y Dauvilliers, CR Baumann, B Carlander, M Bischof, T Blatter, M Lecendreux (2003). CSF hypocretin-1 levels in narcolepsy, Kleine-Levin syndrome, and other hypersomnias and neurological conditions. J Neurol Neurosurg Psychiatry.

[26] Lin L, Faraco J, Li R, Kadotani H, Rogers W, Li X (1999). The sleep disorder canine narcolepsy is caused by a mutation in the hypocretin (orexin) receptor 2 gene. Cell.

[27] American Sleep Disorders Association (1997). ICSD--International Classification of Sleep Disorders (Revised): Diagnostic and Coding Manual. Diagnostic Classification Steering Committee.

[28] Dauvilliers Y, Mayer G, Lecendreux M, Neidhart E, Peraita-Adrados R, Sonka K (2002). Kleine-Levin syndrome: An autoimmune hypothesis based on clinical and genetic analyses. Neurology.

[29] Arnulf I, Zeitzer JM, File J, Farber N, Mignot E (2005). Klein-Levin Syndrome: A systematic review of 186 cases in the literature. Brain.

[30] Arnulf I, Lin L, Gadoth N, File J, Lecendreux M, Franco P (2008). Klein-Levin Syndrome: A systematic study of 108 patients. Ann Neurol.

[31] Harvey AG (2008). Sleep and circadian rhythms in bipolar disorder: Seeking synchrony, harmony, and regulation. Am J Psychiatry.

[32] ViaternaM H, Pinto HL, FW Turek (2005). Principles and Practice of Sleep Medicine. Molecular Genetic Basis for mammalian Circadian Rhythms.

[33] Kilduff TS, Lein EdS, Iglesia Hdl, Sakurai T, Fu Yh, Shaw P (2008). New Developments in Sleep Research: Molecular Genetics, Gene Expression, and Systems Neurobiology. J Neurosci.

[34] King DP, Takahashi JS (2000). Takahashi. Molecular Genetics of Circadian Rhythms in Mammals. Ann Rev of Neurosci.

[35] Franken P, Chollet D, Tafti m (2001). The Homostatic regulation of sleep need is under genetic control. J Neurosci.

[36] Lugaresi E, Medori R, Montagna P, Baruzzi A, Cortelli P, Lugaresi A (1986). Fatal familial insomnia and dys autonomia with selective degeneration of thalamic nuclei. N Engl J Med.

[37] Medori R, Tritschler HJ, LeBlanc A, Villare F, Manetto V, Ying Chen H (1992). Fatal familial insomnia, a prion disease with a mutation at codon 178 of the prion protein gene. N Engl J Med.

[38] Cortelli P, Perani D, Parchi P, Grassi F, Montagna P, De Martin M (1997). Cerebral Metabolism in Fatal Familial Insomnia; Relation to Duration, Neuropathology, and Distribution of Protease-Resistant Prion Protease. Neurology.

[39] Jones CR, Campbell SS, Zone SE, Cooper F, DeSano A, Murphy PJ (1999). Familial advanced sleep-phase syndrome: A short-period circadian rhythm variant in humans. Nat Med.

[40] Taheri S, Mignot E (2002). The genetics of sleep disorders. Lancet Neurol.

[41] Young T, Skatrud J, Peppard PE (2004). Risk factors for obstructive sleep apnea in adults. JAMA.

[42] Riha RL (2006). Genetics Aspects of the Obstructive Sleep Apnea/ Hypopnea Syndrome. Proy Respir Res Basel Karger.

[43] Guilleminault C, Stoohs R, Quera-Salva MA (1992). Sleep-related obstructive and nonobstructive apneas and neurologic disorders. Neurology.

[44] Strohl KP, Gallaugher L, Lynn A, Friedman L, Hill A, Singer JB (2007). Sleep-related epilepsy in the A/J mouse. Sleep.

[45] Abe K, Shimakawa M (1966). Genetic and developmental aspects of sleep walking and teeth grinding. Acta Paedopsychiatrica.

[46] Hublin C, Kaprio J, Partinen M, Heikkilä K, Koskenvuo M (1997). Prevalence and genetics of sleepwalking: a population-based twin study. Neurology.

[47] Behrman R, Kleigman R, Nelson JH (2000). Vegetative disorders (Sleep disorders). Text book of pediatrics.

[48] Magda Lahorgue Nures, Oliviero Bruni (2008). The Genetics of Sleep Disorders in Childhood and Adolescence. Journal de Pediatric (Rio J).

[49] Kaplan HI, Sadock BJ Other disorders of infancy, childhood, or adolescence. Kaplan and Sadock's Synopsis of Psychiatry: Behavioral Sciences, Clinical Psychiatry.

[50] Luciano M, Zhu G, Kirk KM, Gordon SD, Heath AC, Montgomery GW (2007). The genetics of coffee-attributed sleep disturbance. Sleep.

[51] Luisetti M, Seersholm N (2004). Epidemiology of α1-antitrypsin deficiency. Thorax.

[52] Nunes ML, Bruni O (2008). The genetics of sleep disorders in childhood and adolescence. J Pediatr (Rio J).

[53] Campbell SS, Tobler I (1984). Animal sleep, a review of sleep duration across Phylogeny. Neurosci Biobehav Rev.

[54] Hendricks JC (2003). Invited Review: Sleeping flies don't lie: the use of Drosophila melanogaster to study sleep and circadian rhythms. J Appl Physiol.

